# Purification of DNA repair protein complexes from mammalian cells

**DOI:** 10.1016/j.xpro.2021.100348

**Published:** 2021-02-18

**Authors:** Chih-Chao Liang, Martin A. Cohn

**Affiliations:** 1 Department of Biochemistry, Oxford OX1 3QU, UK

**Keywords:** Cell biology, Cancer, Molecular biology, Protein biochemistry, Protein expression and purification

## Abstract

Cells possess multiple DNA repair pathways to tackle a variety of DNA lesions. Often, DNA repair proteins function as large protein complexes. Here, we describe a protocol to purify DNA repair protein complexes from nuclei of mammalian cells. The method permits purification of protein complexes containing stable as well as transiently associated proteins, which subsequently can be identified by mass-spectrometry analysis. This protocol can be applied to uncover the functions and mechanism of DNA repair pathways.

For complete information on the use and execution of this protocol, please refer to Socha et al. (2020).

## Highlights

• Protocol to purify DNA repair protein complexes from a mammalian cell line
• Permits purification of both stable and transiently associated protein subunits
• Protein complexes bound to DNA can also be purified

## Graphical Abstract

**Figure:**
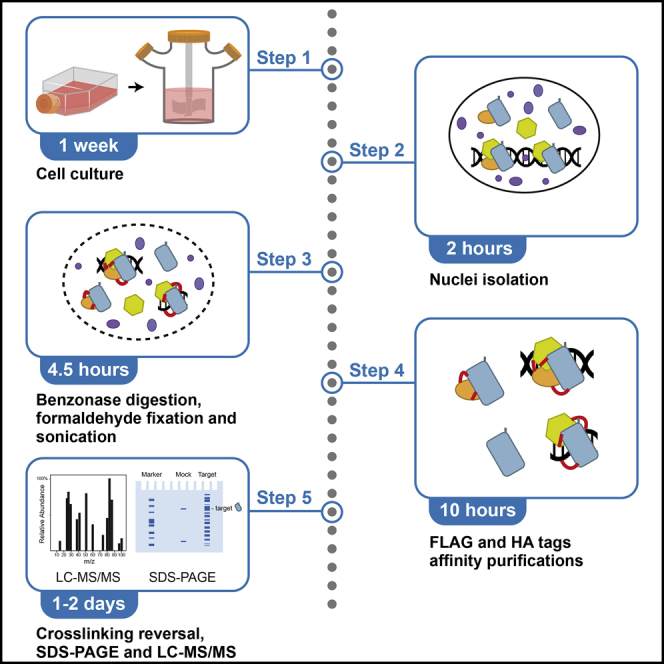

## Before you begin

1. Prepare plenty of buffers in advance. All buffers are filtered with 0.22 μm filters. 0.2 mM PMSF (protease inhibitor) and 2 mM 2-mercaptoethanol (reducing agent) are added freshly to the following buffers immediately before use: Hypotonic buffer, Low-salt nuclear extraction buffer (low-salt NE buffer), High-salt nuclear extraction buffer (high-salt NE buffer) and Buffer A.
2. All purifications are carried out at 4°C.
3. HeLa S3 cells are used in this protocol as they can be grown both as semi-adherent monolayer in dishes or as suspension culture in spinner flasks, enabling large scale culture. Other cell types can be used but will require additional optimization.
4. Methanol-free formaldehyde used is less than 3 months old, preferably less than 1 month.
5. FLAG and HA epitope tags are used for purification as effective antibodies, as well as peptides for elution, are available against both tags for both purification and for detection by immunoblotting analysis. Other tags can be used but will also require additional optimization.

## Key resources table

**Table:** 

REAGENT or RESOURCE	SOURCE	IDENTIFIER
**Experimental models: cell lines**		

Phoenix-A	ATCC	ATCC CRL- 3213
HeLa S3	ATCC	ATCC CCL-2.2

**Antibodies**		

IL-2Rα (7G7/B6)	Merck	05-170
*α*-Tubulin (DM1A) (1:1,000)	Merck	05-829
HA (12CA5) (1:1,000)	Sigma-Aldrich	SAB1305536
FANCD2 (FI17) (1:100)	Santa Cruz	sc-20022
Anti-mouse (horseradish peroxidase conjugated) (1:8,000)	GE Healthcare	NA9310V

**Recombinant DNA**		

pGag+Pol	(Morita et al., 2000)	N/A
pEnv	(Morita et al., 2000)	N/A
pOZ-N	(Nakatani and Ogryzko, 2003)	N/A

**Chemicals, peptides, and recombinant proteins**		

Dulbecco’s modified Eagle’s medium, DMEM	Sigma-Aldrich	D5796
Opti-MEM I reduced serum medium, no phenol red	Thermo Fisher	11058021
Minimum essential medium Joklik’s modified	Sigma-Aldrich	M8028
Fetal bovine serum, FBS	Sigma-Aldrich	F7524
L-Glutamine solution	Sigma-Aldrich	G7513
MEM non-essential amino acid solution (100×)	Sigma-Aldrich	M7145
Penicillin-streptomycin (100×)	Sigma-Aldrich	P4333
Hexadimethrine bromide, Polybrene	Sigma-Aldrich	H9268
Fugene 6	Promega	E2691
Mitomycin C	Sigma-Aldrich	M0440
Benzonase nuclease, 99% purity	Merck	71,206
2-Mercaptoethanol	Sigma-Aldrich	M6250
16% formaldehyde	Thermo Fisher	28906
Phenylmethanesulfonyl fluoride, PMSF	Sigma-Aldrich	P7626
HEPES	Sigma-Aldrich	54457
Glycerol	Sigma-Aldrich	G2025
Tween 20	Sigma-Aldrich	P7949
Sodium dodecyl sulfate, SDS	Sigma-Aldrich	71725
Ethylenediaminetetraacetic acid, EDTA	Sigma-Aldrich	EDS
Glycine	Sigma-Aldrich	50046
FLAG peptide	Sigma-Aldrich	F3290
HA peptide	Sigma-Aldrich	I2149
DL-Dithiothreitol, DTT	Sigma-Aldrich	43815
Iodoacetamide	Sigma-Aldrich	I1149
Trichloroacetic acid, TCA	Sigma-Aldrich	T6399
Dynabeads goat anti-mouse IgG magnetic beads	Invitrogen	11033
Anti-FLAG M2 agarose resin	Sigma-Aldrich	A2220
Protein A Sepharose Cl-4B	Thermo Fisher	GE17-0963-02

**Critical commercial assays**		

Western Lightning Plus-ECL, enhanced chemiluminescence substrate	PerkinElmer	NEL105001EA
SilverQuest silver staining kit	Invitrogen	LC6070

**Other**		

Steritop threaded bottle top filter (PES; 0.22 μm pore size)	Millipore	SCGPT02RE
Glass Dounce homogenizer, 15 mL	Bellco Glass	1984-10015
Econo-Pac chromatography columns	Bio-Rad	7321010
Poly-Prep chromatography columns	Bio-Rad	7311550
MagRack 6 magnetic stand	GE	28948964
Refrigerated centrifuges	N/A	N/A
Sonicator with 3 mm probe	Sonics & Materials	VC 130
Rotating wheel	N/A	N/A
Tube roller	N/A	N/A
Vertical electrophoresis systems	N/A	N/A
Electroblotting systems	N/A	N/A
Horizontal electrophoresis systems	N/A	N/A

## Materials and equipment

### Buffers and other solutions

**Table:** **Phosphate-buffered saline (PBS)** 1,000 mL

Reagent	Final concentration	Amount
NaCl	137 mM	8 g
KCl	2.7 mM	0.2 g
Na_2_HPO_4_	10 mM	1.44 g
KH_2_PO_4_	1.8 mM	0.24 g
CaCl_2_·2H_2_O	1 mM	0.133 g
MgCl_2_·6H_2_O	0.5 mM	0.1 g
Adjust pH to 7.4 with HCl and add ddH_2_O to 1,000 mL. Store at 20–25°C for up to 1 year

**Table:** **Hypotonic buffer** 1,000 mL

Reagent	Final concentration	Amount
1 M HEPES-KOH pH 7.5	10 mM	10 mL
4 M KCl	10 mM	2.5 mL
1 M MgCl_2_	1.5 mM	1.5 mL
Add ddH_2_O to 1,000 mL. Store at 4°C for up to 1 year

**Table:** **Low-salt nuclear extraction buffer (low-salt NE buffer)** 1,000 mL

Reagent	Final concentration	Amount
1 M HEPES-KOH pH 7.5	20 mM	20 mL
4 M KCl	50 mM	12.5 mL
1 M MgCl_2_	1.5 mM	1.5 mL
Glycerol	25%	250 mL
Add ddH_2_O to 1,000 mL. Store at 4°C for up to 1 year

**Table:** **High-salt nuclear extraction buffer (high-salt NE buffer)** 500 mL

Reagent	Final concentration	Amount
1 M HEPES-KOH pH 7.5	20 mM	10 mL
5 M NaCl	1 M	100 mL
1 M MgCl_2_	1.5 mM	0.75 mL
25% Tween 20	1%	20 mL
10% SDS	0.15%	7.5 mL
Glycerol	25%	125 mL
Add ddH_2_O to 1,000 mL. Store at 4°C for up to 1 year

**Table:** **Buffer A** 1,000 mL

Reagent	Final concentration	Amount
1 M Tris-HCl pH 8.0	20 mM	20 mL
5 M NaCl	100 mM	20 mL
1 M MgCl_2_	5 mM	5 mL
25% Tween 20	0.1%	4 mL
Glycerol	10%	100 mL
Add ddH_2_O to 1,000 mL. Store at 4°C for up to 1 year		

**Table:** 

Other solutions	Final concentration	Volume	Note
Hexadimethrine bromide, Polybrene (100×)	0.4 mg/mL in ddH_2_O	10 mL	Store at −20°C for up to 1 year
PMSF (100×)	200 mM phenylmethanesulfonyl fluoride (PMSF) in ethanol	20 mL	Heat to 37°C to dissolve, aliquot to 2 mL per tube and store at −20°C for up to 1 year

## Step-by-step method details

### Generation of a HeLa S3 cell line stably expressing FLAG and HA-tagged FANCD2

This protocol is a modified version of a previously described method developed in the laboratory of Yoshihiro Nakatani (Nakatani and Ogryzko, 2003). As a proof-of-principle, we use human FANCD2 as a target to study its interacting proteins. The human *FANCD2* cDNA was cloned into the pOZ-N retroviral expression vector, which contains a Flag and HA tag at the N-terminus. The pOZ vector contains long terminal repeats to control the integration and expression of target proteins and also a bi-cistronic transcriptional unit for co-expression of interleukin-2 receptor α chain (IL-2Rα) as a selection marker. We normally select for cells or cell clones expressing levels of exogenous proteins comparable with those of endogenous the proteins, to decrease the likelihood of protein-protein interaction being driven by overexpression of the target protein of interest. It should be noted, that while transcription of many genes are cell cycle dependent or regulated in response to cell state or external stimuli, transcription via the described vehicle is more constitutive. Therefore, alternative methods for generating stable or inducible expression can be used. We have successfully used CRISPR/Cas9 to introduce the FLAG-HA tag into the *FANCD2* gene in its chromosomal location, an approach, which might be suitable for other genes (Lopez-Martinez et al., 2019).

**Figure 1:**
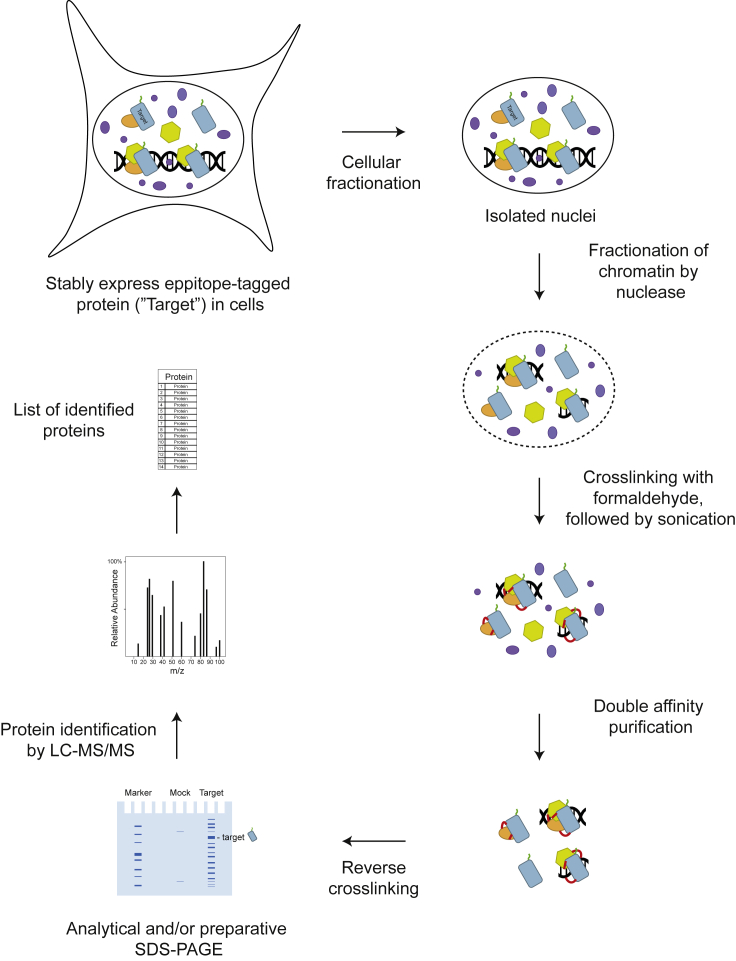
Schematic of protocol

A schematic of this protocol is shown in Figure 1.

### Preparation of retrovirus with packaging cells

**Timing: 30 min**

We use Phoenix-A cells (Phoenix-AMPHO or Phoenix-Amphotropic), a derivative of human 293T cells, for retrovirus packaging, yet supplemented with further Gag, Pol and Env viral proteins via transfection. Phoenix-A cells are grown in DMEM supplemented with 10% FBS in a 37°C incubator with 5% CO_2_. We normally keep the cells in culture for less than 2 months.

1. For one transfection, seed 120,000 cells in one well of a 6-well plate or a 6-cm dish (roughly 10% confluency).

**CRITICAL:** It is important to plate an appropriate amount of cells. Too few cells will yield low titer virus stock, and too many cells will deplete the nutrition in the medium, which negatively affects the viral transduction to follow.

2. Prepare the transfection mixture in two separate microcentrifuge tubes. Tube No 1: Target plasmids and two helper plasmids

**Table:** 

100 μL	DMEM medium (without serum and antibiotics) or opti-MEM
1 μg	pOZ-N-FANCD2 plasmids or desired plasmids
0.5 μg	pGag+Pol
0.5 μg	pEnv

**Table:** Tube No 2: Transfection agent

100 μL	DMEM medium (without serum and antibiotics) or opti-MEM
6 μL	Fugene 6

3. Pipette the DNA mixture in Tube No 1 into Tube No 2, invert 5 times, and incubate at 20–25°C for 15–30 min.
4. Pipette the whole transfection mixture drop-wise into the well with Phoenix-A cells.
5. Gently rock the plate to help the transfection mixture disperse into the medium.
6. Let the cells grow for 66–72 h.
7. Harvest the retrovirus and pass the supernatant through a 0.22 μm filter.

**CRITICAL:** The color of the medium should be red/orange but not yellow. The Phoenix-A cells can be harvested and lysed for assessing expression level of protein of interest by SDS-PAGE and immunoblotting with specific antibodies.

### Preparation of IL-2Rα antibody conjugated magnetic beads

**Timing: 30–60 min and 30 min**

For using the IL-2Rα antibody conjugated magnetic beads in cell selection in the next section, the preparation is done in a sterile environment, e.g., laminar flow hood.

8. Prepare 30 mL of 0.1% FBS in PBS (referred to as PBS/FBS).
9. Aliquot 1.8 mL of Dynabeads Goat Anti-Mouse IgG Magnetic Beads into three 1.5 mL microcentrifuge tubes (600 μL each).
10. Place the tubes on a magnetic stand for 2 min and gently remove the supernatant. Remove the tubes from the magnetic stand, and gently resuspend the beads with 1 mL PBS/FBS.
11. Repeat the magnetic capturing and washing (step 10) twice. After the last wash, gently resuspend the beads with 600 μL PBS/FBS.
12. Dissolve 200 μg of lyophilized anti-IL-2Rα antibody (whole vial) with 600 μL of PBS/FBS. Transfer 20 μL to a separate tube, this is the “input” sample for later SDS- PAGE analysis of samples.
13. Add 200 μL of anti-IL-2Rα antibody to each tube of beads.
14. Incubate the tubes on a slow rotator in the cold room for 12–16 h.
15. Place the tubes on a magnetic stand for 2 min and gently transfer the supernatant to a 15-mL tube. This sample is considered “flow though.” Remove the tubes from the magnetic stand, and gently resuspend the beads with 1 mL PBS/FBS.
16. Repeat the magnetic capturing and washing (step 15) three times, though without keeping the supernatant.
17. For each tube, resuspend the beads in 1 mL PBS/FBS supplemented with 50% glycerol. These are the “coupled beads.” Aliquot the beads in 200 μL per 1.5 mL microcentrifuge tubes. Store the beads at −20°C.
18. Analyze “input,” “flow through” as well as “coupled beads” by SDS-PAGE followed by Coomassie stain. IgG should be present in “input” and “coupled beads” samples, but not in the “flow through” sample.

***Note:*** Excessive freeze-thaw cycles will lead to dissociation of the IL-2Rα antibody from the magnetic beads.

### Transducing HeLa S3 cells with retrovirus and sorting for transduced cells

**Timing: 10–15 min and 1.5 h**

DMEM supplemented with 2.5%–10% FBS is used when HeLa S3 cells are grown as semi-adherent monolayer in cell culture dishes in an incubator at 37°C and 5% CO_2_. We normally keep the cells in culture for less than 2 months.

19. Supplement the retrovirus stock with 4 μg/mL of Hexadimethrine bromide (polybrene).
20. Detach the HeLa S3 cells by treating the cells with PBS supplemented with 2 mM EDTA, and pellet down roughly 200,000 cells in a 15 mL tube (188 × *g*, 20°C, 5 min).
21. Resuspend the cells with the virus stock and plate them in a 9 cm dish.
22. Let the cells grow in the virus stock overnight (less than 24 h).

***Note:*** Polybrene is toxic to the cells. It is important to not leave the cells in medium with retrovirus and polybrene for longer than 24 h. Longer incubation can lead to increased cell death.

23. Collect and centrifuge the supernatant (188 × *g*, 20°C, 5 min).

***Note:*** Normally there will be a portion of cells that remain partially adhered.

24. Discard the supernatant, resuspend the cell pellet with fresh supplemented DMEM, and plate the cells back in the 9 cm dish. Allow to grow in an incubator at 37°C and 5% CO_2_ till the HeLa S3 cells reach 60%–80% confluency (66–72 h).
25. Detach the transduced HeLa S3 cells by treating the cells with PBS supplemented with 2 mM EDTA, and centrifuge (188 × *g*, 20°C, 5 min).

***Note:*** If trypsin was used, it is important not to over-trypsinize the cells. IL-2Rα, which is localized on the cell surface and used for sorting in the following step, could be damaged leading to ineffective sorting.

26. Aspirate the supernatant and resuspend the cells with 1 mL fresh supplemented DMEM in a 1.5 mL microtube.
27. Add 10 μL of 50% slurry IL-2Rα antibody conjugated magnetic beads (prepared in steps 8–18) to the cells and leave the tube on a roller at 20–25°C for 1 h.

**CRITICAL:** The incubation is to allow the IL-2Rα antibody conjugated magnetic beads to properly bind to the recombinant IL-2Rα on the cell surface. Do not leave the tube rolling for more than 1 h.

28. Place the tube on a magnetic stand for 2 min, and gently remove the supernatant. Remove the tube from the magnetic stand, and gently resuspend the cells with the beads with 1 mL fresh DMEM.

**CRITICAL:** Do not pipette vigorously or vortex the tube. The IL-2Rα antibody conjugated magnetic beads could detach from the cells due to shear force.

29. Repeat the magnetic capturing and washing (step 28) twice.
30. Resuspend the cells with 1 mL supplemented DMEM and plate them into a 9 cm dish containing 9 mL of supplemented DMEM.

Alternatives: the cells can be diluted with conditioned medium and plated on 96-well plate as single cell in each well to select for individual clones.

**Figure 2:**
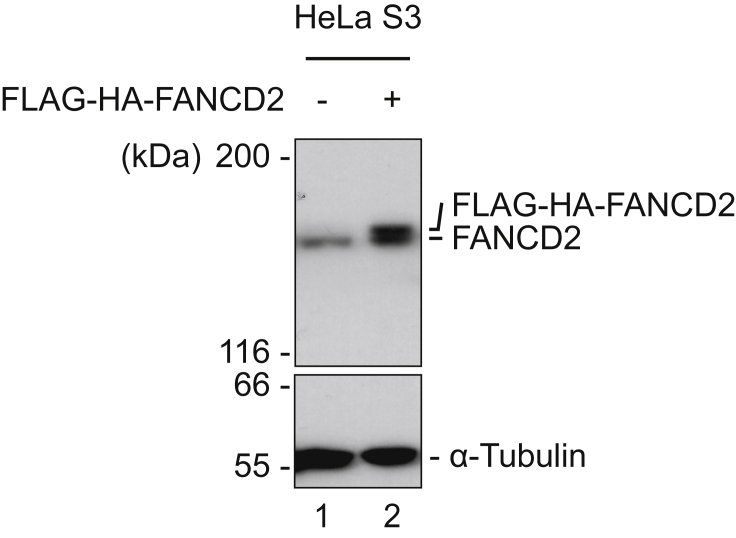
Expression of FLAG-HA-tagged FANCD2 in HeLa cells FLAG-HA-tagged FANCD2 is expressed at levels comparable to endogenous FANCD2 in HeLa S3 cells. Immunoblot analysis.

***Note:*** Due to the bi-cistronic expression of both the target gene and IL-2Rα, the number of magnetic beads bound to the cell surface could be used to assess the expression level of target gene prior to immunoblot analysis. It should be noted, that the ideal level of expression of the FLAG-HA-tagged protein is typically such that the level is comparable to that of the endogenous protein (Figure 2). Overexpression of the tagged protein can lead to abnormal cellular localization, spurious protein-protein interaction, and other artifacts.

### Preparation of nuclear extract from HeLa S3 cells

**Timing: 4.5 h**

This part of the protocol is a modified version of an original protocol of cellular fractionation developed by the laboratory of Robert Roeder (Dignam et al., 1983). We introduced a DNA digestion step with Benzonase nuclease to reduce chromatin mediated interaction, and to permit purification of complexes associated with chromosomes. Furthermore, we introduced a formaldehyde fixation step adapted from other cell systems (Aparicio et al., 2004) to preserve weak protein-protein interactions, which otherwise could be lost during the purification. HeLa S3 cells are grown as suspension cultures in spinner flasks in an incubator at 37°C (no CO_2_ regulation required) using Joklik’s Modified MEM supplemented with 2.5% FBS, 2 mM L-glutamine, non-essential amino acids (100× dilution) and penicillin-streptomycin (100× dilution). HeLa S3 cells are normally grown in DMEM supplemented with 2.5% FBS in dishes as described in the previous section. To set up larger cultures in spinner flask, HeLa S3 cells from an 80% confluent 15 cm dish are detached and centrifuged as described in the previous section. The cells are resuspended in 50 mL supplemented Joklik’s medium and grown in a 125 mL spinner flask in a 37°C incubator. The color of the medium should change to orange (but not yellow) over time, and the culture is expanded by adding in equal volume of fresh supplemented Joklik’s medium to the same flask (or transferred to a larger spinner flask). The volume of the culture will double each day until the desired scale is reached. We optimized each step in the following nuclear extract preparation and purification of protein complexes with 1 L starting culture. Once optimal purification results were achieved, we scaled the experiment to 6 L culture. The following steps indicate volume used for 1 L starting culture.

***Note:*** The fractionation procedure is carried out at 4°C or on ice throughout. All the buffers are pre-chilled to 4°C and freshly supplemented with 0.2 mM PMSF and 2 mM β-ME.

31. Treat the cells with 160 ng/mL Mitomycin C for 16 h.

***Note:*** This step is optional. The Mitomycin C treatment is to induce DNA interstrand crosslinks (ICLs) in the genome and to activate the ICL repair pathways. Alternative treatment, or using unperturbed cells, could be relevant.

32. Harvest the cells by centrifuging the culture (1,800 × *g*, 4°C, 5 min) and discard the supernatant.
33. Wash the cell pellet by resuspending it with 50 mL cold PBS, centrifuge (1,800 × *g*, 4°C, 5 min) and discard the supernatant.
34. Equilibrate the cell pellet with hypotonic buffer by resuspending the cell pellet with 2 mL of hypotonic buffer. Centrifuge (1,800 × *g*, 4°C, 5 min) and discard the supernatant.

**CRITICAL:** Do not leave the cells incubated in the hypotonic buffer at this step. Otherwise, the cells will start swelling, which will affect the efficiency of fractionation.

35. Resuspend and incubate the pellet in 4 mL (twice pellet volume) of hypotonic buffer on ice for 10 min.

***Note:*** The cells will be swollen at the end of the incubation. It is important to observe the changes of cell size under a microscope to confirm the efficiency of hypotonic treatment.

36. Transfer the solution into a pre-chilled glass Dounce homogenizer on ice.
37. Use a B type, tight pestle, with a clearance of 20–56 μm, to press up and down promptly and steadily for 10–20 cycles on ice.

**CRITICAL:** Dounce homogenizers can vary slightly, and different cell types will require varying amount of cycles. It is important to collect samples of cells before and after homogenization, and examine these under a microscope to evaluate the efficiency of cell membrane rupture, which should be above 90%. Continue homogenization until sufficient cell breakage is achieved. Insufficient rupture will lead to the cytoplasmic fraction from unbroken cells contaminating the nuclear fraction. On the other hand, excessive homogenization will result in breakdown of the nuclear envelope, leading to the loss of soluble nuclear proteins from nuclear fractions.

38. Centrifuge the solution to pellet nuclei (3,300 × *g*, 4°C, 15 min).
39. Remove the top lipid layer by inserting a clean pipette tip into the solution, which the lipid layer will adhere to.
40. **Cytoplasmic fraction**: Collect the supernatant as the cytoplasmic fraction and supplement with 11% of the volume of 10× S-100 buffer.

**Table:** S-100 buffer (10× stock) 100 mL

Reagent	Final concentration	Amount
1 M Tris-HCl pH 7.3	300 mM	30 mL
4 M KCl	1.4 M	35 mL
1 M MgCl_2_	30 mM	3 mL
Add ddH_2_O to 100 mL. Store at 4°C for up to 1 year
41. Centrifuge the cytoplasmic fraction (17,000 × *g*, 4°C, 30 min) and remove the lipid layer as described above.

***Note:*** The cytoplasmic fraction can be dialyzed against the desired buffer if needed for subsequent purification. In this protocol, we focus on isolating nuclear protein complexes.

42. **Nuclear fraction with Benzonase digestion and formaldehyde fixation**: Resuspend the nuclei in 1 mL (half of pellet volume) low-salt NE buffer supplemented with 0.01% Triton X-100 and 75 unit/mL Benzonase, and leave it incubating on ice for 30 min.

***Note:*** The amount of Benzonase added and the incubation time can be adjusted if needed. Digestion of chromosomal DNA can be monitored by agarose gel electrophoresis.

43. Supplement the mixture with formaldehyde to final concentration of 1% and incubate at 20–25°C for 4 min to crosslink the nuclei.

***Note:*** The nuclei solution will turn opaque and slightly clumpy after crosslinking.

44. Quench the formaldehyde by adding 150 μL (1/20 volume) of 2.5 M Glycine pH 7.5 to the mixture.
45. Centrifuge the mixture (3,000 × *g*, 4°C, 10 min) and discard the supernatant.
46. Wash the crosslinked nuclei by resuspending the nuclei with 10 mL (10 times pellet volume) of PBS, centrifuge (3,000 × *g*, 4°C, 10 min) and discard the supernatant.
47. Repeat step 46 twice to wash the nuclei two more times.
48. Resuspend the crosslinked nuclei with 1 mL (same pellet volume) of high-salt NE buffer.

***Note:*** The protein complexes are now crosslinked and stabilized, so we can use extraction condition with high stringency without disrupting protein complexes.

49. Sonicate the crosslinked nuclei for 20 min (with 1 s on/off intervals) at 4 W power in an ice/water slurry bath.

***Note:*** The sample would normally turn clear after sonication, and the pellet from centrifugation (step 52) is small.

***Note:*** In addition to the Benzonase digestion, this sonication step renders chromatin DNA to smaller fragments.

50. Leave the sonicated nuclei incubating on ice for 30 min.
51. Dilute the lysate 5-fold with 8 mL low-salt NE buffer supplemented with 125 mM NaCl and 1% Tween 20.

***Note:*** This dilution step is to lower the concentration of SDS from 0.15% to 0.03% and NaCl from 1 M to 300 mM to make it compatible with anti-FLAG M2 agarose used in the next stage.

52. Centrifuge the solution (17,000 × *g*, 4°C, 20 min) and collect the supernatant as nuclear extract.

**Pause point:** The protein complexes should be preserved by formaldehyde crosslinking (step 43). The nuclear extract can be snap-frozen using liquid nitrogen and stored at −80°C for purification later.

### Immunoaffinity purification

Sequential affinity purifications usually yield proteins of higher purity or, in this case, more pure protein complexes. However, the handling during sequential purification usually leads to loss of some interacting proteins, especially those with weaker affinities. With our modified nuclear extraction protocol, we are able to perform the sequential affinity purifications with higher stringency to reduce non-specific binding to the antibodies and matrixes while retaining the specific protein complexes. In this protocol, we purify FANCD2 sequentially via the FLAG and HA tags engineered at its N-terminus. Though using this protocol, we typically observe negligible level of contaminants (Socha et al., 2020), we recommend performing a parallel “mock” purification from cells not expressing a FLAG-HA-tagged protein, to assess the level of background binding proteins.

The purification is carried out at 4°C or on ice throughout.

### FLAG tag affinity purification

**Timing: 5 h**

53. Add 250 μL bed volume of anti-FLAG M2 agarose beads to the 10 mL nuclear extract, and incubate the tube on a rotator at 10 rpm for 1.5–2 h at 4°C.

***Note:*** The amount of anti-FLAG M2 agarose used in the purification is determined empirically by monitoring the depletion of FANCD2 from the nuclear extract during purification. We use 250 μL bed volume of anti-FLAG M2 agarose for a 1 L HeLa S3 culture. When targeting different proteins with distinct expression level, it will require optimization.

***Note:*** Prior to use, the anti-FLAG M2 agarose beads are washed with 5 column volume (c.v.) of 100 mM glycine pH 2.5, 10 c.v. of ddH_2_O and 10 c.v. of buffer A. The glycine wash is to remove antibodies not crosslinked to the agarose matrix.

***Note:*** Anti-FLAG purification is performed in batch mode to improve the binding efficiency.

54. Centrifuge the mixture (1,000 × *g*, 4°C, 5 min) and collect the supernatant as flow- through fraction of anti-FLAG purification step.
55. Resuspend the beads with 1 mL Buffer A and transfer the beads to a clean gravity-flow chromatography column.
56. Carefully apply 10 mL Buffer A to wash the beads and repeat the washing step two more times.

***Note:*** The washing of the beads is more efficient if allowing most of the buffer to drip through before applying more buffer.

57. Cap the outlet and apply 250 μL (equal volume of the anti-FLAG beads) of Buffer A supplemented with 0.5 mg/mL FLAG peptide. Seal the top with parafilm and leave the column on a roller at 4°C for 1 h.

***Note:*** We perform the elution in batch because it allows FLAG peptide to compete away FLAG-tagged FANCD2 from anti-FLAG beads efficiently, helping to keep the volume of eluate low, thus more concentrated.

58. Collect the FLAG eluate and repeat the elution (step 57) once more.

***Note:*** An extra elution can be performed, while we usually observe a decrease in concentration.

59. Pool all FLAG eluates.

### HA tag affinity purification

**Timing: 5 h**

Overall, the HA tag affinity purification is carried out similarly to the FLAG tag affinity purification described in the previous section with minor modifications. We generally use less anti-HA Sepharose beads comparing to the anti-FLAG M2 agarose used in the previous section. We use 0.5 mg/mL HA peptide for elution.

60. Add 60 μL bed volume anti-HA Sepharose beads to the FLAG eluate from the previous step, and incubate the tube on a rotator at 10 rpm for 2 h at 4°C.

***Note:*** Similarly to step 53, the necessary amount of anti-HA Sepharose needs to be determined empirically.

***Note:*** Prior to use, the anti-HA beads are washed and equilibrated as described for the anti-FLAG M2 agarose beads in step 53, the second Note.

***Note:*** We perform the anti-HA purification in batch because it provides better binding efficiency.

61. Centrifuge the mixture (1,000 × *g*, 4°C, 5 min) and collect the supernatant as the flow-through fraction of the anti-HA purification step.
62. Resuspend the beads with 1 mL Buffer A and transfer the beads to a clean gravity-flow chromatography column.
63. Carefully apply 5 mL Buffer A to wash the beads and repeat the washing step two more times.

***Note:*** The washing of the beads is more efficient if allowing most of the buffer to drip through before applying more buffer.

64. Cap the outlet and apply 60 μL (equal volume to the anti-HA beads) of Buffer A supplemented with 0.5 mg/mL HA peptide. Seal the top with parafilm and leave the column on a roller at 4°C for 1 h.

***Note:*** We perform the elution in batch because it allows HA peptide to compete away HA-tagged FANCD2 from anti-HA beads efficiently, which helps to keep the volume of eluate low with good concentration.

Alternatives: the protein complexes can be eluted from the anti-HA Sepharose matrix with 0.1 M Glycine pH 2.5, followed by pH neutralization of the eluate by adding 1/5 volume of 1.5 M Tris pH 8.8 immediately after collecting the eluate. It is faster and more efficient using Glycine for elution. However, it could also elute proteins bound non-specifically to the matrix.

65. Collect the HA eluate and repeat the elution (step 64) once more.

***Note:*** An extra elution can be performed, though we usually observe a decrease in the overall concentration as a result.

### Reversion of formaldehyde crosslinks and sample preparation for mass spectrometry analysis

**Timing: 3.5 h**

We reverse the formaldehyde crosslinks and alkylate our sample with Iodoacetamide prior to mass spectrometry analysis. Different mass spectrometry processes may require samples being processed differently after reversal of formaldehyde crosslinks.

66. Supplement the HA eluate with 0.1% SDS and 5 mM DTT and incubate the sample at 95°C for 20 min to reverse the formaldehyde crosslinks.

Alternatives: the HA eluate can be supplemented with SDS-PAGE loading buffer, incubate the samples at 95°C for 20 min, and load the samples onto SDS-PAGE followed by Coomassie or silver staining for analysis.

67. Dilute the sample ten-fold with milli-Q water supplemented with 5 mM DTT and cool the sample on ice for 5 min.

***Note:*** The dilution is to prevent SDS from precipitating on ice at higher concentration. If some precipitants are formed, one could slightly warm up the sample till the precipitants are dissolved again.

68. Supplement the sample with 15 mM Iodoacetamide and incubate the sample at 20–25°C protected from light for 20 min.

***Note:*** Iodoacetamide is light sensitive. It is important to protect the sample from light.

69. Supplement the sample with 10 mM of DTT and incubate the sample at 20–25°C protected from light for 15 min.

***Note:*** The concentration of DTT at this step is 15 mM.

70. Supplement the sample with 10% TCA by mixing equal volume of sample with 20% TCA and incubate the sample on ice 2–16 h, protected from light.
71. Centrifuge the sample (17,000 × *g*, 4°C, 30 min) and discard the supernatant.
72. Rinse the pellet with −20°C 100% acetone gently, centrifuge the sample (17,000 × *g*, 4°C, 10 min) and discard the supernatant carefully.
73. Let the pellet air-dry at 20–25°C.
74. Analyze the precipitated protein complexes by MS/MS.

## Expected outcomes

**Figure 3:**
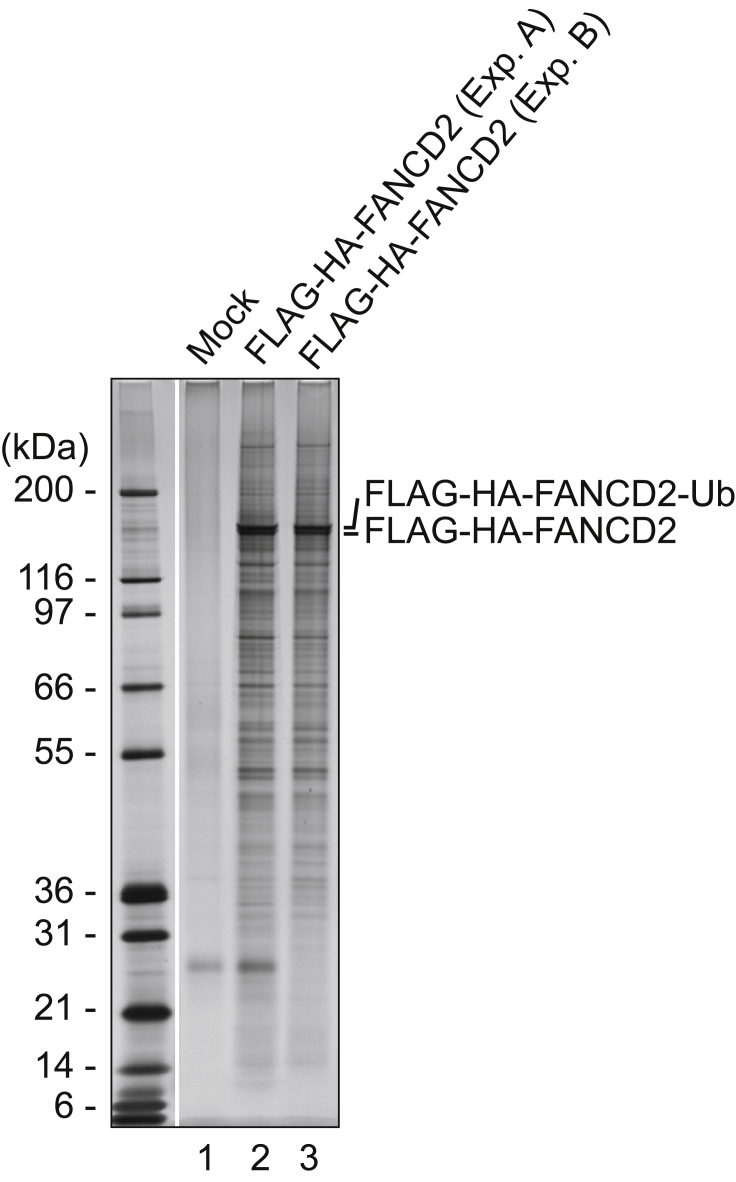
Purification of FANCD2-containing protein complexes from HeLa cells is highly reproducible FANCD2-containing complexes purified from two independent experiments were resolved by SDS-PAGE and visualized using silver stain. Lane 2, small scale purification (experiment A). Lane 3, large scale purification (experiment B). Comparable profiles of polypeptides are observed. A mock purification (lane 1) contains essentially no polypeptides. The clean mock purification and the reproducibility of purified complexes indicate the robustness of the protocol. Parts of this figure (marker lane and lanes 1 and 3) are identical to previously published data, and are reprinted with permission (Socha et al., 2020).

This protocol allows purification of FLAG- and HA-tagged protein containing complexes. The optimized formaldehyde crosslinking protocol allows co-purification of weakly interacting proteins. We typically analyze replicate purifications with SDS-PAGE followed by silver or Coomassie staining to test for reproducibility (step 66). An example of this is shown in Figure 3, which show two independent purifications of FANCD2 complexes. One of the two purifications is identical to a previously published purification (Socha et al., 2020). The gel image is shown again here, side-by-side with an independent purification, as an example of how robust and reproducible the method is.

## Limitations

This protocol was optimized to purify FANCD2 containing complexes. FANCD2 is a nuclear protein, which is recruited to chromatin during replication and/or upon DNA damage. The formaldehyde crosslinking step was introduced after nuclei isolation. If the protein of interest is cytoplasmic or soluble nuclear protein, the timing of formaldehyde crosslinking needs to be further optimized. The FLAG and HA tags on the N-terminus of FANCD2 permits purification of this protein, however other proteins might require C-terminal tagging to permit purification, and to preserve function of the fusion protein. We discuss this potential issue in the troubleshooting section problem 5.

## Troubleshooting

### Problem 1

Low expression of target gene in HeLa S3 cells after retrovirus transduction (step 19).

### Potential solution

The quality of retrovirus packaging in Phoenix-A cells is very important. Good transfection efficiency is essential. Other transfection agents can also be used but require further optimization.

The color of medium, which reflects the nutrition level and the pH, on the day of harvesting retrovirus is critical. If the cellular density of the Phoenix-A cells for transfection is too high or the retrovirus is harvested too late, the nutrition in the medium would be depleted, which cannot support the HeLa S3 cells during the retrovirus transduction step. If the medium is yellow on the day of harvesting retrovirus, one can try to replace the retrovirus containing medium after 6 h or supplement with some fresh medium.

Aggregation of the HeLa S3 cells during incubation with anti-IL-2Rα antibodies conjugated magnetic beads can lead to contamination by non-transduced cells. Additional rounds of cell selection with anti-IL-2Rα antibodies conjugated magnetic beads can be applied a few days after the first round of sorting to select for true-positive cells.

### Problem 2

Contamination of cytoplasmic proteins in nuclear fraction or vice versa (steps 37, 40, and 42).

### Potential solution

We routinely check the efficiency of our cellular fractionation by immunoblotting using antibodies against cytoplasmic proteins, e.g., α-Tubulin, and nuclear or chromatin proteins, e.g., Lamin B and histones.

The first wash with hypotonic buffer (step 34) is designed to increase the efficiency of the hypotonic treatment in step 35. However, it is important to centrifuge cells immediately after resuspension, follow the incubation times carefully, and to monitor the cells under a microscope. If different cell type is used, the time of incubation and number of strokes of the Dounce homogenizer should be optimized.

As stated in the step-by-step section, there can be variation between Dounce homogenizers. It is important to examine the before and after samples carefully under a light microscope. If the nuclear envelope is disrupted, try reducing the number of strokes. Likewise, if plasma membranes are not disrupted sufficiently, increase the number of strokes. When collecting the cytoplasmic fraction after centrifugation of nuclei, try to remove as much solution as possible without disturbing the nuclei pellet.

### Problem 3

Inconsistent fragmentation of chromatin DNA by Benzonase digestion and sonication (step 42).

### Potential solution

This protocol relies on Benzonase to digest chromosomal DNA to smaller fragments. We supplemented the buffer with 0.01% Triton X-100 to improve the accessibility of chromatin to Benzonase. We tried to keep the concentration of detergent as low as possible at this step to avoid disrupting any protein-protein interaction. One could try to further increase the concentration of detergent or increase the amount of Benzonase used at this step.

### Problem 4

Insufficient extraction of target protein (step 52).

### Potential solution

Sonication (step 49) helps the rupture of nuclei and further shear the Benzonase digested DNA to smaller fragments. The crosslinked nuclei should be sonicated over a time-course to identify optimal condition for protein extraction. We monitor the extraction of target protein by immunoblotting. Both the power level and sonication time can be adjusted. While the nuclei are crosslinked and protein complexes are preserved at step 43, it is important to avoid warming up the solution during sonication.

### Problem 5

Low recovery of target protein during immunoaffinity purification (steps 58 and 65).

### Potential solution

This protocol was developed to purify FANCD2-containing protein complexes. Different proteins of interest might provide different accessibility for purification. First, confirm that the tags used for purification are accessible during non-crosslinked native condition. It is possible that formaldehyde crosslinking affects the accessibility of the tags by stabilizing some interacting proteins near the tags. Extending the linker between tag and protein of interest or reducing the degree of crosslinking by lowering the formaldehyde concentration or shortening the time of crosslinking at step 43 can help. C-terminal tagging rather than N-terminal tagging can also be considered.

We optimized our protocol with the use of formaldehyde as the crosslinking agent. However, there are limitation due to its small size (about 2 Å) and amino group preferences (Hoffman et al., 2015; Metz et al., 2004), which could lead to loss of true interacting partners identified in the subsequent mass spectrometry analysis. Similar to conventional chromatin immunoprecipitation (ChIP), other crosslinking agents, such as EGS (ethylene glycol bis(succinimidyl succinate)) or DSG (di(N-succinimidyl glutarate)) can be used in substitute or in combination of formaldehyde to improve crosslinking efficiency (Singh et al., 2019; Tian et al., 2012; Zeng et al., 2006). If other crosslinkers are used, timing of crosslinking, quenching and extraction will require further optimization. It should be noted that not all crosslinks are reversible, which can affect the subsequent mass spectrometry analysis.

On the other hand, crosslinks between proteins can be used to successfully map direct protein-protein interactions via mass spectrometry (Mintseris and Gygi, 2020; O'Reilly and Rappsilber, 2018).

The affinity between different tags and their binding counterparts also varies. Reducing the stringency of binding and washing condition at step 53 and onwards are options. It is possible to extend the time of binding at step 53. Alternative tags can be considered.

## Resource availability

### Lead contact

Further information and requests for resources and reagents should be directed to and will be fulfilled by the lead contact, Martin A. Cohn (martin.cohn@bioch.ox.ac.uk).

### Materials availability

This study did not generate new unique reagents.

### Data and code availability

This study did not generate new data or code.
